# Impact of Rainfall on Aquatic and Terrestrial Species Monitoring Using Riverine Environmental DNA

**DOI:** 10.1002/ece3.74259

**Published:** 2026-09-01

**Authors:** Chen Xu, Kei Nukazawa

**Affiliations:** ^1^ Interdisciplinary Graduate School of Agriculture and Engineering University of Miyazaki Miyazaki Japan; ^2^ Department of Civil and Environmental Engineering, Faculty of Engineering University of Miyazaki Miyazaki Japan

**Keywords:** environmental DNA metabarcoding, generalized linear mixed model, Permutational Multivariate Analysis of Variance, rainfall, vertebrate

## Abstract

Water‐based environmental DNA (eDNA) can simultaneously detect aquatic and terrestrial species during watershed‐scale eDNA biodiversity monitoring. However, detecting terrestrial species remains a challenge because of their complex environmental effects. This study targeted vertebrate eDNA transported from land to rivers through surface runoff during rainfalls in three rivers within the Kiyotake River watershed, Japan. We compared the relative detection indices of different taxonomic groups under rainfall and non‐rainfall conditions and determined how environmental factors (e.g., discharge and filtration volume) influence sample‐level alpha and beta diversity. The results showed that mammals and amphibians were detected more frequently after rainfall, whereas fish showed lower detection frequencies due to reduced filtration volumes caused by filter clogging. Generalized linear mixed model analyses revealed that river discharge had significant negative effects on the detection number, whereas filtration volume had a significant positive effect. Furthermore, Permutational Multivariate ANOVA indicated that filtration volume and rainfall occurrence significantly affected beta diversity. Our findings highlight the importance of filtration volume for detecting alpha and beta diversity and demonstrate that eDNA‐based biomonitoring remains effective during rainfall events, offering practical guidance for enhancing biodiversity assessments at the watershed scale. Such approaches may provide an efficient tool for detecting not only aquatic species, but also terrestrial vertebrates that are otherwise difficult to survey using conventional field methods.

## Graphical abstract

Environmental DNA (eDNA) has enabled simultaneous monitoring of aquatic and terrestrial vertebrates, yet the influence of rainfall on detection patterns remains poorly understood. In this study, we sampled river water in the Kiyotake catchment in Japan and compared vertebrate detection patterns under rainfall and non‐rainfall conditions. As a result, rainfall increased the detection efficiency of terrestrial and semi‐aquatic species but reduced those of fish species, highlighting the importance of sampling design in watershed‐scale eDNA monitoring.

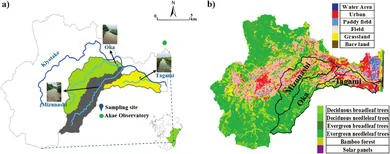

## 1 Introduction

Global climate change and human activity have accelerated biodiversity loss, leading to the significant degradation of ecosystem services (Cardinale et al. 2012). Environmental DNA (eDNA) enables the development and implementation of effective monitoring strategies for timely, evidence‐based conservation. eDNA, comprising genetic material (e.g., skin and feces) shed by organisms and retained in environmental substrates (e.g., water, soil, and air) (Guldberg Frøslev et al. 2022; Redondo et al. 2020; Yang et al. 2023). It offers several advantages, including low cost, rapidity, and non‐invasiveness (Dyson et al. 2024; Fediajevaite et al. 2021; Suren et al. 2024). In addition, eDNA metabarcoding, which combines next‐generation sequencing and universal primers, facilitates large‐scale biodiversity surveying (Afzali et al. 2021; Taberlet et al. 2012; Valentini et al. 2016).

Vertebrate eDNA research has mainly focused on aquatic species (Huang et al. 2022; Ruppert et al. 2019; Sahu et al. 2023; Takahashi et al. 2023). For terrestrial species, a range of sampling strategies and substrates (e.g., air, soil, mineral licks, feeding traces, and hair) has been employed. For example, fecal samples have been used to detect carnivores (Meyer et al. 2020), while DNA extracted from wildflowers has been used to identify arthropods and birds (Thomsen and Sigsgaard 2019). Tailoring eDNA sampling protocols for specific taxonomic groups or ecological contexts may improve the comprehensiveness and reliability of biodiversity assessments. However, identifying suitable environmental substrates for terrestrial vertebrate eDNA is time‐consuming and resource‐intensive (Gogarten et al. 2020; Seeber and Epp 2022). These limitations may restrict the range of species detected (Newton et al. 2025). Moreover, considerable uncertainty remains regarding the ecology of terrestrially derived eDNA, including its origin, physicochemical properties, transport mechanisms, and environmental fate (Guthrie et al. 2024; Hassan et al. 2022).

Terrestrial species can release DNA into the aquatic environments through activities such as drinking and swimming (Rodgers and Mock 2015; Ushio et al. 2017), providing a theoretical basis for water‐based eDNA approaches for the simultaneous and rapid detection of terrestrial and aquatic species. Such approaches have been applied across various ecosystems, such as forests, urban areas (Martinelli Marín et al. 2024; Reji Chacko et al. 2023; Saenz‐Agudelo et al. 2022), and watersheds ranging from small streams to the Amazon River (Coutant et al. 2021; Macher et al. 2021). However, in water‐based eDNA biodiversity monitoring, the detection rates of terrestrial species are generally lower than those of aquatic species (Ryan et al. 2022; Zhang et al. 2023). This discrepancy largely reflects continuous DNA shedding by aquatic organisms (e.g., fish), while terrestrial species exhibit limited eDNA deposition due to the spatially and temporally intermittent interactions between terrestrial species and water bodies (De Souza et al. 2016). As a result, target species may remain undetected, even when present near sampling locations (Harper et al. 2019). Furthermore, the detectability of eDNA in aquatic systems is influenced by environmental factors such as pH, water temperature, and flow distance (Mauvisseau et al. 2022; Nukazawa et al. 2018; Seymour et al. 2018; Strickler et al. 2015). This highlights the need to use water‐based approaches to elucidate the effect of environmental factors on the detection of aquatic and terrestrial species.

However, eDNA detection in both terrestrial substrates and aquatic environments has inherent limitations (Cowgill et al. 2024). Water is the most commonly used eDNA substrate for biodiversity assessing (Newton et al. 2025), due to its ability to connect terrestrial and aquatic habitats, as well as upstream and downstream areas, allowing the evaluation of species diversity across broader spatial scales (Deiner et al. 2016; Yang et al. 2023). In our previous study on a specific terrestrial mammal, we confirmed that eDNA could be transported into rivers via surface runoff during rainfall events (Xu and Nukazawa 2025). This will help improve the detection efficiency of large‐scale assessments of terrestrial mammalian diversity. In addition to terrestrial mammals, rainwater collected after precipitation events in forested ecosystems contains a high diversity of invertebrate and microbial taxa (Ladin et al. 2021; Macher et al. 2023). In riverine ecosystems, post‐rainfall river water samples show an increased microbial diversity (Bae et al. 2022). However, studies on vertebrates in such settings are limited. In a previous study using eDNA samples, the number of fish species detected in river water decreased after rainfall, whereas the number of mammalian species detected increased (Staley et al. 2018). This indicates that rainfall had distinct effects on the detection of vertebrate species with different ecological habits. Moreover, different rainfall patterns (e.g., intensity and duration) may affect species detection. For instance, heavy‐rainfall periods are less suitable for fish detection than light rainfall periods. Compared to peak rainfall, rainfall duration is more conducive to the detection of terrestrial mammals (Condachou et al. 2025; Osathanunkul and Suwannapoom 2024; Xu and Nukazawa 2025). These findings highlight the need to further investigate how rainfall patterns influence the eDNA detection of vertebrates, including fish and mammals.

This study developed an eDNA‐based approach for detecting aquatic and terrestrial biological communities in riverine stormwater collected after rainfall events in the Kiyotake River catchment in Miyazaki Prefecture, Japan. Furthermore, we evaluated the effects of different rainfall patterns on species richness and community composition across sub‐catchments with contrasting land use types, providing new insights into the use of aquatic eDNA to enable the simultaneous and rapid detection of different taxonomic groups, such as fish and mammals.

## 2 Materials and Methods

### 2.1 Sampling and eDNA Extraction

**FIGURE 1:**
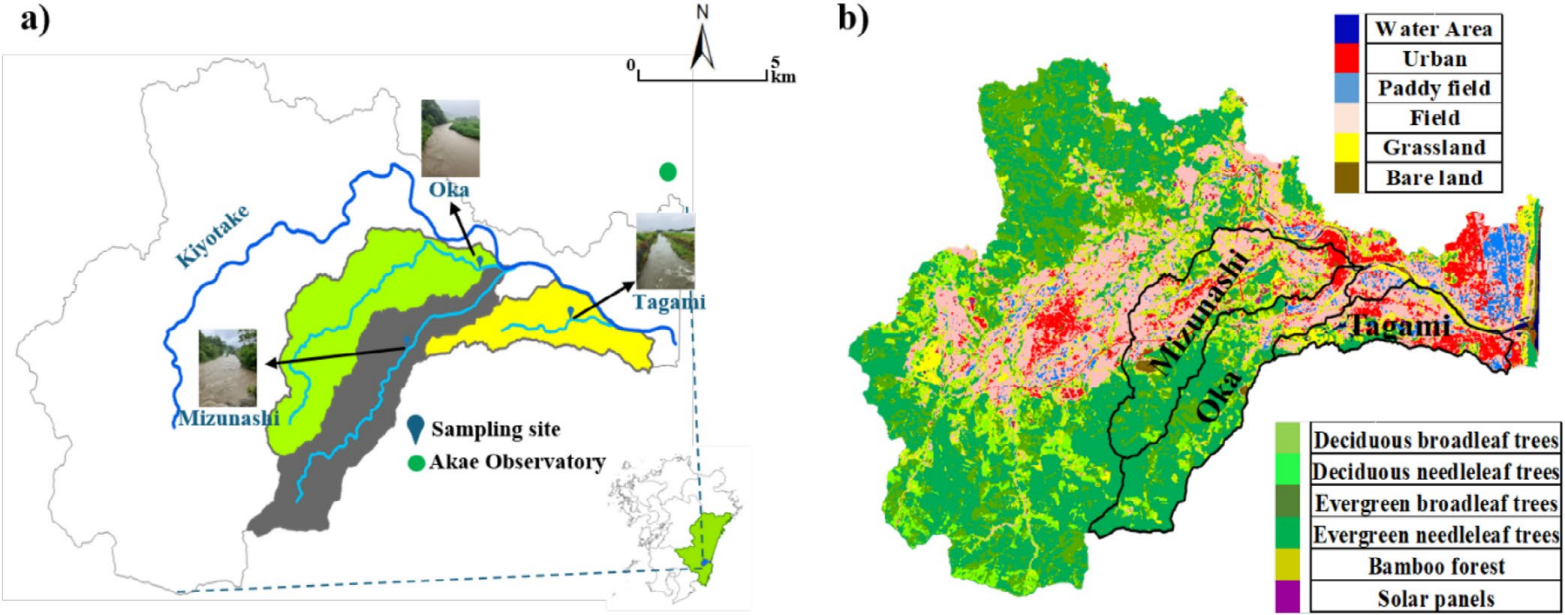
(a) Sampling sites in the Mizunashi River, Oka River, and Tagami River (blue mark) and a weather station (green point) in the Kiyotake River Basin, located in southwestern Japan. (b) Land use distribution map. The maps were processed using ArcGIS, with geographic layers obtained from the Geospatial Information Authority of Japan (https://nlftp.mlit.go.jp/ksj/). Sampling sites were plotted based on GPS coordinates. The watershed boundary was delineated using ArcGIS. All other graphic elements were prepared by the authors. Photos of the river after rainfall were taken by the authors.

Sampling, filtration, and eDNA extraction procedures have been described in detail in our previous study (Xu and Nukazawa 2025). Briefly, sampling was conducted during both rainfall and non‐rainfall events at three tributaries (the Mizunashi River, Oka River, and Tagami River) within the Kiyotake River watershed in Miyazaki Prefecture, southwestern Japan (Figure 1). Rainfall‐event sampling was conducted on May 12, September 14, and November 29, 2022, and June 2, 2023. To investigate temporal variation in eDNA detection, two sampling sessions (S1 and S2) were performed approximately 2 h apart on May 12, 2022, and June 2, 2023. During rainfall events, two replicate 1‐L water (R1 and R2) samples were collected at each sampling site. Non‐rainfall‐event sampling was conducted on November 28, 2022, and August 8, 2024, during which a single 1‐L water sample was collected from each site. Environmental parameters, including water temperature, pH, electrical conductivity (EC), and turbidity, were measured. The corresponding data are shown in Table 1. However, due to technical issues, environmental parameters were not measured on November 28, 2022, and EC was not measured on June 2, 2023. All water samples were filtered through glass fiber filters with a pore size of 0.7 μm. If a filter became clogged during filtration, it was replaced with a new filter to continue processing the sample. Filtration was terminated if the second filter also became clogged, and the remaining water sample was discarded. After filtration, all filters were stored at −20°C until DNA extraction. Environmental DNA was extracted from samples using the DNeasy PowerSoil Kit (Qiagen, Hilden, Germany) according to the manufacturer's instructions, with a final elution volume of 100 μL. The extracted DNA was stored at −80°C. Because the DNeasy PowerSoil Kit was discontinued, samples collected on August 8, 2024, were extracted using its successor, the DNeasy PowerSoil Pro Kit (Qiagen, Hilden, Germany), following the manufacturer's protocol. All equipment used during sampling, filtration, and extraction was decontaminated with bleach or sterilized prior to use to minimize the risk of DNA contamination.

**TABLE 1:** Results of water quality analysis. “Sampling1” and “Sampling2” indicate the first and second samplings of the day, with Sampling2 conducted 2 h after the start of Sampling1.

Sample	Turbidity (ppm)	Electrical conductivity (μS/cm)	pH	Water temperature (°C)	Discharge (m^3^/s)	Filtration volume (L)
2022_5_Mizunashi_Sampling1	19.30	53.60	7.96	14.90	2.84	1.00 (1.00)
2022_5_Mizunashi_Sampling2	25.38	61.10	7.91	17.70	2.84	0.95 (0.93)
2022_5_Oka_Sampling1	174.52	76.70	7.44	19.70	4.35	0.63 (0.42)
2022_5_Oka_Sampling2	46.73	71.30	7.18	18.40	4.35	0.89
2022_9_Oka_Sampling1	63.25	55.70	7.70	21.60	4.11	1.00 (1.00)
2022_9_Tagami_Sampling1	16.72	67.30	7.11	24.20	0.77	1.00 (1.00)
2022_11_Mizunashi_Sampling1	45.12	98.20	7.87	18.00	1.54	0.61 (0.69)
2022_11_Tagami_Sampling1	138.54	39.80	7.18	19.00	0.51	0.35 (0.38)
2022_11_Mizunashi_no‐rainfall	Not measured				0.26	1.00
2022_11_Oka_no‐rainfall					0.33	1.00
2022_11_Tagami_no‐rainfall					0.07	1.00
2023_6_Mizunashi_Sampling1	285.82	—	7.72	17.90	6.01	0.30 (0.35)
2023_6_Mizunashi_Sampling2	110.31	56.60	6.61	18.00	6.01	0.31 (0.58)
2023_6_Oka_Sampling1	314.34	—	7.47	20.90	11.23	0.31 (0.31)
2023_6_Oka_Sampling2	112.33	67.10	6.09	21.00	11.23	0.70 (0.56)
2023_6_Tagami_Sampling1	12.77	—	6.90	21.00	2.12	1.00 (1.00)
2023_6_Tagami_Sampling2	7.43	76.70	5.98	21.00	2.12	1.00 (1.00)
2024_8_Mizunashi_no‐rainfall	1.19	13.37	7.31	25.80	0.52	1.00
2024_8_Oka_no‐rainfall	0.14	16.36	8.48	28.00	0.73	1.00
2024_8_Tagami_no‐rainfall	1.99	25.50	7.54	29.80	0.13	1.00

*Note:* “–” indicates data not recorded. Measurements were not taken after the no‐rainfall event in November 2022. Values in parentheses indicate the filtration volume for replicate samples.

### 2.2 eDNA Metabarcoding

Using the DNeasy PowerClean Pro Cleanup Kit (Qiagen, Hilden, Germany), the template was purified to remove the PCR inhibitors. The concentration of the DNA solution was determined using the Synergy LX (Agilent Technologies, USA) in combination with the QuantiFluor dsDNA System (Promega, USA). Vertebrate DNA was comprehensively detected via high‐throughput sequencing using a two‐step tailed PCR approach targeting the mitochondrial 12S rRNA gene region. The forward primer YAGAACAGGCTCCTCTAG and reverse primer TTAGATACCCCACTATGC were used because of their low species‐level primer bias, high specificity, and reproducibility (Collins et al. 2019; Riaz et al. 2011). Moreover, previous studies reported that replacing the initial thymine (T) in the forward primer with pyrimidine (Y) enables broader binding across vertebrate taxa (Kocher et al. 2017). The first‐round PCR (1st PCR) was performed in eight replicates, with each 10‐μL PCR mixture containing 1.0 μL of 10× Ex Buffer, 0.8 μL of deoxynucleotide triphosphates (dNTPs, 2.5 mM each), 0.5 μL each of 10‐μM forward: 5′‐ACACTCTTTCCCTACACGACGCTCTTCCGATCT‐YAGAACAGGCTCCTCTAG‐3′ and reverse: 5′‐GTGACTGGAGTTCAGACGTGTGCTCTTCCGATCT‐TTAGATACCCCACTATGC‐3′ (underline indicates the Illumina adapter sequence) primer, 2.0 μL of template DNA, 0.075 μL of ExTaq HS (5 U/μL; TaKaRa), and 5.125 μL of nuclease‐free water. The thermal cycling conditions were as follows: initial denaturation at 94°C for 2 min; 30 cycles at 94°C for 30 s, 50°C for 30 s, and 72°C for 30 s; and a final extension at 72°C for 5 min. When the concentration of the initial DNA solution was low, the number of cycles increased to 35. The PCR products were purified using VAHTS DNA Clean Beads (Vazyme, China) at a 1.5× ratio to the reaction volume. The purified products were used as templates for the second round of PCR. A negative control was prepared using nuclease‐free water as the template. The 2nd PCR process was performed as a single reaction to attach the adapter sequences. Each 10‐μL reaction mixture included 5.0 μL of 2× PCR Buffer for KOD FX Neo, 2.0 μL of dNTPs (2 mM each), 0.5 μL each of 5‐μM forward: 5′‐AATGATACGGCGACCACCGAGATCTACAC[index2]ACTCTTTCCCTACACGACGC‐3′ and reverse: 5′‐CAAGCAGAAGACGGCATACGAGAT[index1]GTGACTGGAGTTCAGACGTGTG‐3′ (based on the Illumina Adapter Sequences Document: https://support.illumina.com/content/dam/illumina‐support/documents/documentation/chemistry_documentation/experiment‐design/illumina‐adapter‐sequences‐1000000002694‐14.pdf) primer, 1.0 μL of pooled 1st‐PCR products, 0.2 μL of KOD FX Neo (1.0 U/μL; TOYOBO, Japan), and 0.8 μL of nuclease‐free water. The PCR program was as follows: 94°C for 2 min; 12 cycles at 98°C for 30 s, 60°C for 30 s, 68°C for 30 s; and 68°C for 5 min. When the concentration of the 1st PCR product was too high, the number of cycles was reduced to 10. The 2nd PCR products were also purified using VAHTS DNA Clean Beads (Vazyme, China) at a 1.5× ratio to the reaction volume. The concentration of the final libraries was measured using Synergy H1 (Agilent Technologies, USA) with the QuantiFluor dsDNA System, and library quality was assessed using a Fragment Analyzer with the dsDNA 915 Reagent Kit (Agilent Technologies, USA). Finally, sequencing was conducted using the MiSeq system with the MiSeq Reagent Kit v3 (Illumina, USA), under 2 × 300‐bp paired‐end conditions.

Raw read data were cleaned by removing primer sequences using the FASTX‐Toolkit (ver. 0.0.14). Low‐quality sequences with quality scores of < 20 were removed using Sickle (ver. 1.33), and reads shorter than 40 bases pairs were discarded. Paired‐end reads were merged using the FLASH script (ver. 1.2.11) with a minimum overlap of 10 bases. For species identification, the dada2 plugin in Qiime2 (ver. 2024.2) was used to remove the chimeric and noisy sequences. Representative sequences and an Amplicon Sequence Variant (ASV) table were generated, and the representative sequences were subjected to a Basic Local Alignment Search Tool (BLAST) against the nucleotide database (National Center for Biotechnology Information) to identify species. The identification criterion was a coverage > 90%, and only the results with the highest identity was used. The identity thresholds for species‐level identification were set as follows: > 98% for species, > 95% for genera, > 90% for families, and > 85% for orders (Veron et al. 2023). When the identity index was the same, the optimal result was selected as the reference index, based on the *E*‐value and coverage. In cases where there was incongruence between BLAST hits, the ASV(s) would be assigned to a higher rank (i.e., genus, family, or class) to provide a consensus assignment (Chung et al. 2024). ASVs with read counts below 100 were removed to control for potential minor contaminations, sequencing errors, and other sources of noise.

Because the samples were collected from freshwater rivers, based on the fish database (https://www.fishbase.se), fish species that exclusively inhabited marine environments were excluded from the analysis. Human sequences were also excluded because of human activity in the sampling area. Species not distributed in Japanese fields were likewise excluded. The excluded results are retained in the Supporting Information but were not used in subsequent analyses.

One ASV was assigned to *Homo sapiens* , although it may have originated from *Mus musculus* . Although *Mus* is often regarded as a laboratory contaminant, its widespread presence in natural environments warranted the retention of this ASV in the analysis.

### 2.3 Data Analysis

#### 2.3.1 Relative Detection Index (RDI)

To evaluate the impact of rainfall on species detection, we calculated the relative detection index (RDI) of the total and taxonomic groups for each sample. As a comprehensive species inventory for each river is currently lacking, the total number of species detected across all samples was used as a proxy for the regional species pool. Based on this, the total RDI was defined as the proportion of species detected relative to the total number of species detected in the river. The RDI for the taxonomic group was defined as the proportion of species detected within that group in the sample relative to the total number of species detected in the river.

Because this study spanned an entire year and encompassed all four seasons, seasonal variations could confound the effects of rainfall on species detection. In particular, birds exhibit strong seasonal migratory behavior, and their detection frequency may therefore be influenced more by seasonal patterns than by rainfall. Moreover, because of the limited taxonomic resolution of the primers used in this study, most bird sequences could not be reliably identified to the species level, making it difficult to determine whether the detected taxa were migratory. To minimize the influence of seasonal factors and enhance the robustness of the analysis, birds were excluded from the RDI and community structure calculations. In contrast, although some fish species also migrate for spawning, they remain in freshwater rivers during certain life stages; therefore, seasonal variations do not preclude their detection.

#### 2.3.2 Relationship Between Detection Number and Environmental Factors

Generalized linear mixed models (GLMMs) with a Poisson distribution were used to assess the relationships between the number of species detected in the samples (detection numbers) and environmental factors, including turbidity, pH, water temperature, filtration volume, and discharge. Because EC was not recorded in June 2023, it was excluded from the model. The cumulative rainfall during the 3 h prior to sampling was used as an explanatory variable. Rainfall data were obtained from the Akae Meteorological Observatory, the nearest weather station to the study area (available at the Japan Meteorological Agency website: https://www.data.jma.go.jp/obd/stats/etrn/index). River discharge following rainfall events was estimated using a distributed hydrological model (Mineda et al. 2023). Because stream discharge could not be directly estimated for the Tagami River, discharge was first estimated for the Kiyotake River and subsequently converted based on the ratio of the Tagami River watershed area to that of the Kiyotake River watershed. Variance inflation factors (VIFs) were calculated using the R “car” package to verify multicollinearity. Variables with VIFs > 5 were iteratively removed until all variables had VIF values less than 5. Turbidity was excluded from this process. The final models were constructed using the R “glmmTMB” package (Brooks et al. 2017) with species richness as the response variable; pH, water temperature, filtration volume, flow rate, and cumulative rainfall over the preceding 3 h as explanatory variables; and sampling site and sampling month as random effects. After modeling, the “DHARMa” package was used to conduct an overdispersion test of the model based on simulated residuals (dispersion = 0.978, *p* = 0.912). All analyses were conducted using R (ver. 4.3.2, R Core Team 2023).

#### 2.3.3 Community Structure Analyses

To evaluate the effect of rainfall conditions on community composition, we calculated β‐diversity based on species presence/absence data, using the Sørensen dissimilarity index (Baselga 2010). Specifically, used the “betapart” package to compute total dissimilarity (*β*_sor_) (Baselga and Orme 2012), turnover (*β*_sim_), and nestedness (*β*_sne_), where *β*_sor_ = *β*_sim_ + *β*_sne_. Heatmaps were generated using Origin (2025) software (OriginLab, USA) to visualize diversity patterns. To investigate the influence of environmental variables on the directional variation in community structure, principal coordinate analysis (PCoA) was conducted based on the Sørensen dissimilarity index. Using the “envfit()” function (permutations = 999) in the vegan package, with a fixed random seed to ensure reproducibility, we fitted the three environmental variables that were fully recorded (i.e., discharge, filtration volume, and rainfall) onto the ordination plots, and only those with significant effects were displayed, thus allowing for a clear visualization of the direction and strength of their influence on community composition.

To assess the effects of rainfall occurrence (categorical variable: presence/absence) and filtration volume (continuous variable) on community dissimilarity (*β*_sor_, *β*_sim_, and *β*_sne_), we used Permutational Multivariate Analysis of Variance (PERMANOVA). PERMANOVA was performed using 999 permutations with the “permute” and “vegan” packages. A fixed random seed was set to ensure reproducibility. The analysis was implemented as a marginal‐effects model (i.e., by = “margin” in the “adonis2()” function), enabling the independent evaluation of each explanatory variable. As *β*_sor_ is a non‐Euclidean distance measure, it was square‐root‐transformed prior to analysis; *β*_sim_ and *β*_sne_ were adjusted using the Lingoes correction to meet the assumptions of PERMANOVA. All analyses were conducted using R (ver. 4.3.2, R Core Team 2023).

## 3 Results

### 3.1 eDNA Metabarcoding

No amplification was detected in the nuclease‐free water control. A total of 1,952,639 sequences were obtained from the 33 eDNA samples. One sample collected from the Oka River in June 2023 showed no detection. Among the sequences, 93.15% had a quality score > 30. After removing the chimeric and noisy sequences, 812,677 reads remained. Following the removal of non‐vertebrate species, humans, and environmental contaminants, 668,629 reads remained and were classified as fish, birds, amphibians, or mammals. Fish accounted for the largest proportion (66.03%), followed by mammals (16.64%), amphibians (13.30%), and birds (4.03%). In total, 57 taxa were detected, with 38 taxa identified at the species level, representing 66.7% of the total.

**FIGURE 2:**
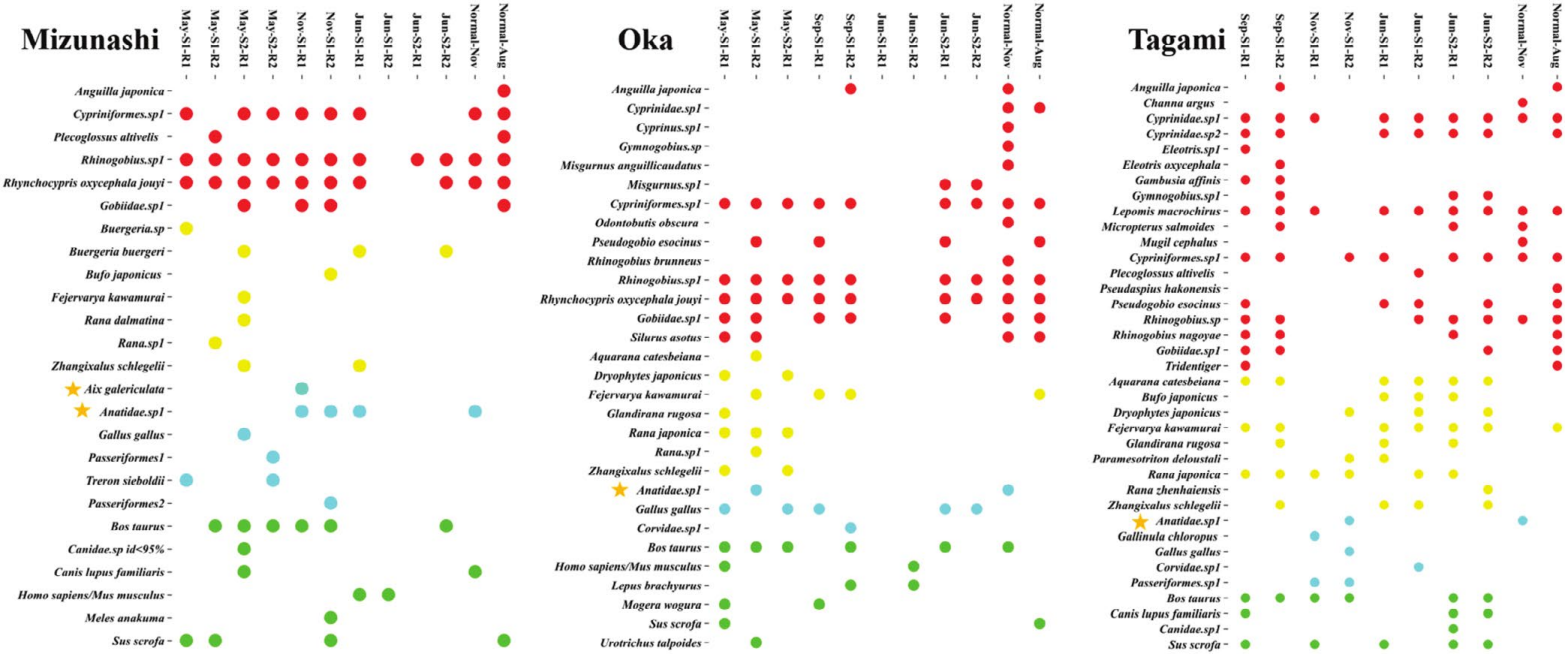
Detection results for each river. Circles indicate presence, with red, yellow, blue, and green representing the detections of fish, amphibians, birds, and mammals, respectively. “S1” and “S2” indicate the first and second sampling sessions on each sampling day, respectively. “R1” and “R2” represent sample replicates 1 and 2. “Normal” indicates non‐rainfall conditions, with sampling dates on November 28, 2022, and August 8, 2024. The others are rain events. The sampling dates during rainfall events were May 12, September 14, and November 29, 2022, as well as June 2, 2023. Filled stars indicate waterbirds.

Figure 2 shows the detection results for each river (for the complete dataset, see Data S1). In the Mizunashi River, 25 taxa were detected, including 6 fish species, 7 amphibians, 6 birds, and 6 mammals. In the Oka River, 30 taxa, including 14 fish, 7 amphibians, 3 birds, and 6 mammals, were identified. The Tagami River had the highest taxonomic richness, with 37 taxa, specifically 19 fish, 9 amphibians, 5 birds, and 4 mammals. The Oka River and Tagami River showed a higher dominance of fish species. Most of the detected mammalian taxa were domesticated or human‐associated species (e.g., cattle, pigs, and dogs). The wild mammals detected in this study included *Meles anakuma* , *Mogera wogura* , *Urotrichus talpoides* , *Lepus brachyurus* , *Canidae* spp. (domestic and raccoon dogs), and *Sus scrofa* (wild boars and domestic pigs). Amphibian taxa were also predominantly detected after rainfall events. Except *Paramesotriton deloustali* , a salamander species that is not native to Japan but has been introduced through the pet trade, all remaining taxa belong to the order Anura. The bird taxa included both migratory and resident birds. However, because of the limitations of the primers, they could not be identified at the species level. Except *Gallus gallus* (domestic chicken), all other wild bird species were detected at very low frequencies except for *Gallus gallus* (domestic chicken).

### 3.2 RDI and Detection Number

**FIGURE 3:**
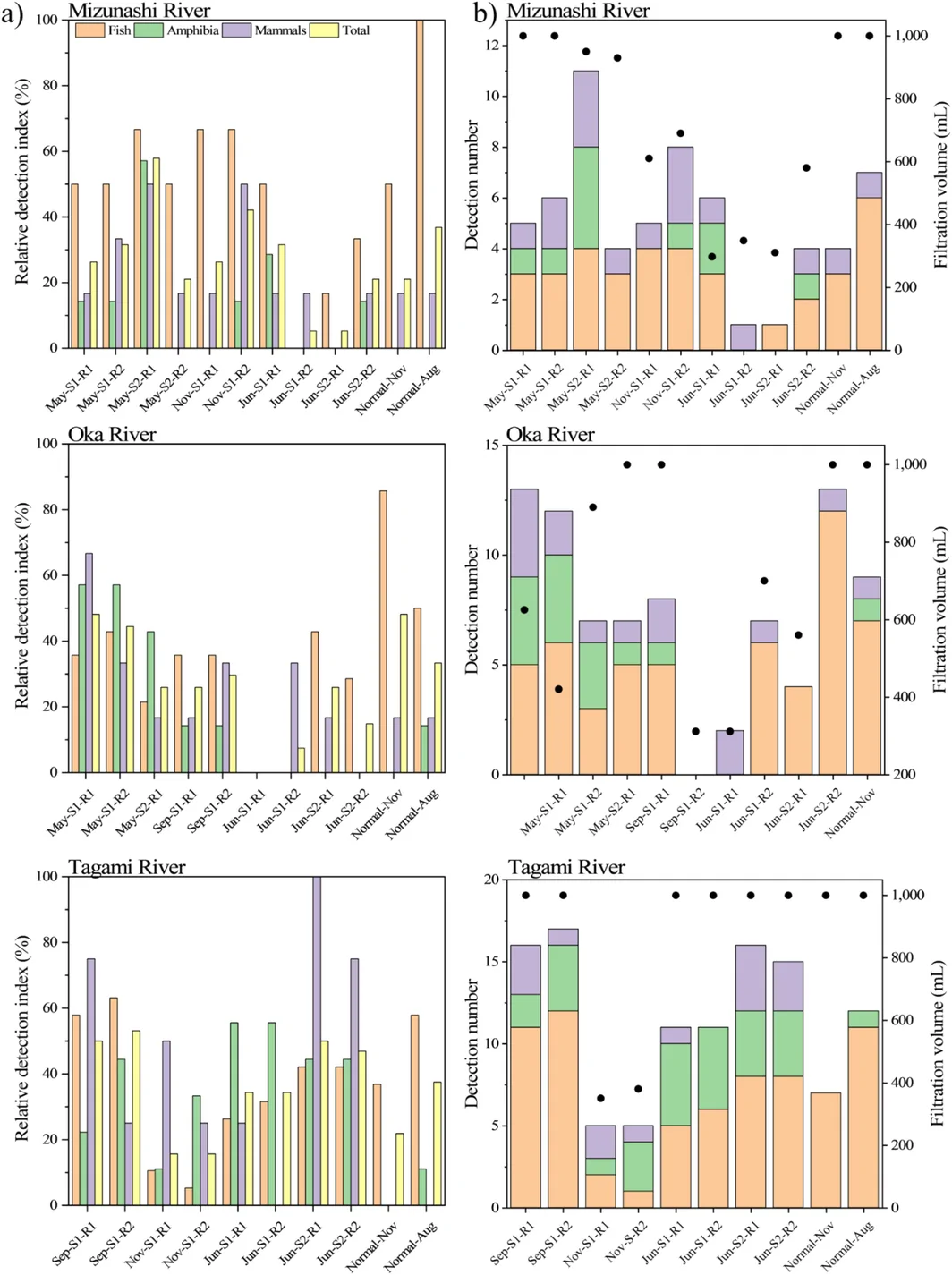
Relative detection index (RDI) of each taxonomic group and overall RDI at each sampling site. “S1” and “S2” indicate the first and second sampling sessions on each sampling day, respectively. “R1” and “R2” represent sample replicates 1 and 2, respectively. Detection results for each river. Circles indicate presence, with red, yellow, blue, and green representing the detections of fish, amphibians, birds, and mammals, respectively. “S1” and “S2” indicate the first and second sampling sessions on each sampling day, respectively. “R1” and “R2” represent sample replicates 1 and 2. “Normal” indicates non‐rainfall conditions, with sampling dates on November 28, 2022, and August 8, 2024. The others are rain events. The sampling dates during rainfall events were May 12, September 14, and November 29, 2022, as well as June 2, 2023. “Total” indicates the overall RDI for all taxonomic groups.

Figure 3a shows the RDI of each sample. For the Mizunashi River, the RDI ranged from 5.26% to 57.89%. The two sampling events without rainfall yielded RDI of 21.05% and 36.84%. In the Oka River, the RDI ranged from 0% to 48.15%. The two non‐rainfall sampling events had RDI of 48.15% and 33.33%. For the Tagami River, the RDI varied between 15.63% and 53.13%, with the two non‐rainfall sampling events exhibiting RDI of 21.88% and 37.50%. Regarding different taxonomic groups, the RDI in the Mizunashi River ranged from 0% to 100% for fish, 0% to 57.14% for amphibians, and 0% to 50% for mammals. In the Oka River, the RDI ranged from 0% to 85.71% for fish, 0% to 57.14% for amphibians, and 0% to 66.67% for mammals. Meanwhile, for the Tagami River, the RDI ranged from 5.26% to 63.16% for fish, 0% to 55.56% for amphibians, and 0% to 100% for mammals.

Figure 3b shows the filtration volume and number of detected taxa for each sample. Differences in the number of detected taxa were observed among all samples, including between replicate samples. This variation was greatest in the Mizunashi River (3–7 taxa), followed by the Oka River (1–3 taxa) and Tagami River (0–1 taxa). When the filtration volumes were similar between the replicate samples, the difference in the number of detected taxa was generally reduced to 0–1. The number of detected fish taxa increased in the Tagami River on the sampling dates, with two sampling events conducted on the same day. The number of detected mammalian taxa increased in the Mizunashi River but decreased in the Oka River.

### 3.3 Relationship Between Detection Number and Environmental Factors

Table 2 presents the results of GLMMs. Filtration volume had a significant positive effect on the number of detections, whereas the discharge had significant negative effects on the detection number. Rainfall, water temperature, and pH had no significant effect on the number of detections.

**TABLE 2:** Relationships between rainfall, water temperature, pH, filtration volume, and discharge with the detection number analyzed using generalized linear mixed models.

Total				
	Estimate	Std. Error	*z*	Pr(>|*z*|)
(Intercept)	3.934	1.121	3.510	< 0.001
Rainfall	−0.009	0.008	−1.179	0.238
Temperature	−0.006	0.028	−0.214	0.831
pH	−0.261	0.134	−1.949	0.051
Volume	0.709	0.347	2.045	**0.041**
Discharge	−0.095	0.037	−2.543	**0.011**

*Note:* Bold values indicate *p* < 0.05.

### 3.4 Community Structure

**FIGURE 4:**
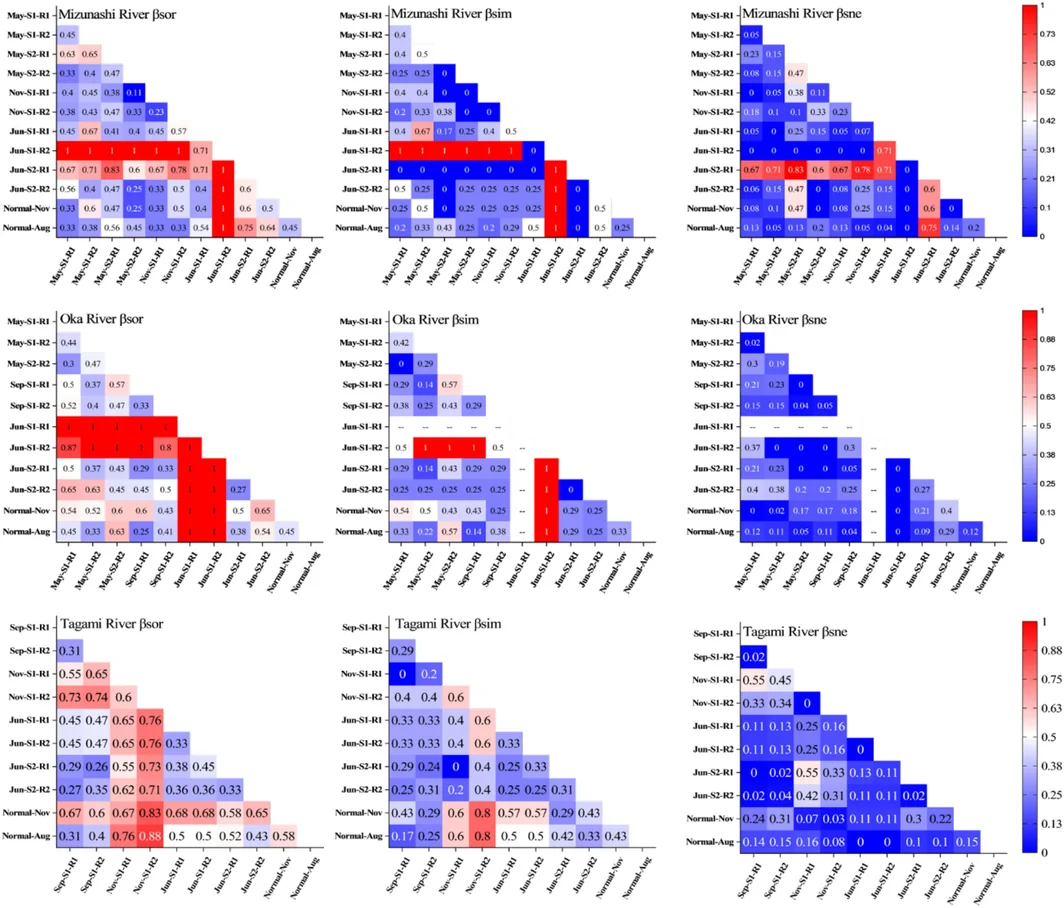
*β*_sor_, *β*_sim_, and *β*_sne_, calculated based on the Sørensen dissimilarity index, for three sampling sites. “S1” and “S2” indicate the first and second sampling sessions on each sampling day, respectively. “R1” and “R2” represent sample replicates 1 and 2, respectively. “Normal” indicates non‐rainfall conditions, with sampling dates on November 28, 2022, and August 8, 2024. The others are rain events. The sampling dates during rainfall events were May 12, September 14, and November 29, 2022, as well as June 2, 2023.

Figure 4 shows the *β*_sor_, *β*_sim_, and *β*_sne_ results, calculated based on Sørensen dissimilarity, for the three sampling sites. The average *β*_sor_, *β*_sim_, and *β*_sne_ values for each river were as follows: the Mizunashi River: *β*_sor_ = 0.56, *β*_sim_ = 0.35, and *β*_sne_ = 0.21; the Oka River (excluding the sample with no detectable sequences): *β*_sor_ = 0.56, *β*_sim_ = 0.42, and *β*_sne_ = 0.14; the Tagami River: *β*_sor_ = 0.54, *β*_sim_ = 0.37, and *β*_sne_ = 0.17.

**FIGURE 5:**
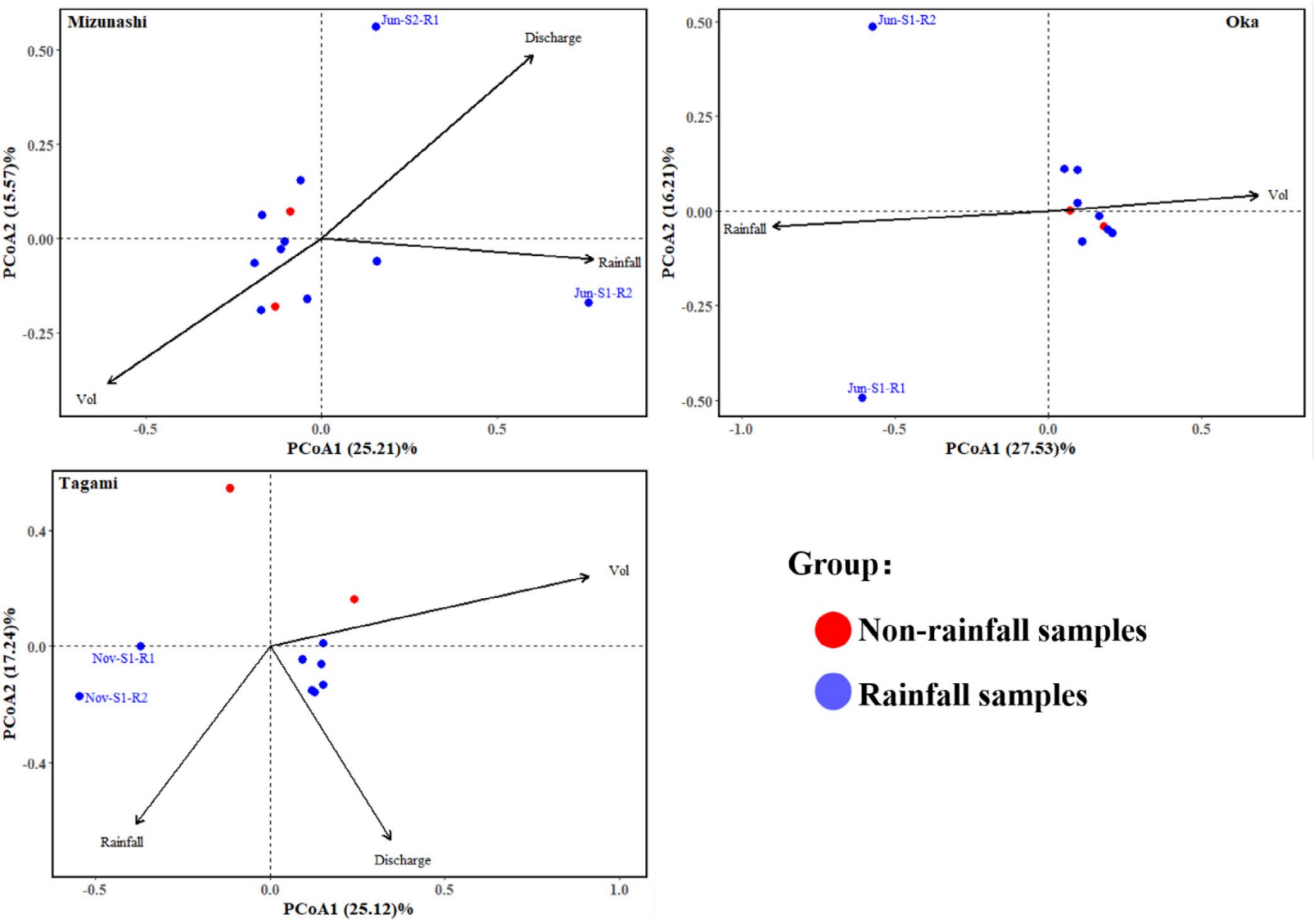
Principal coordinates analysis of eDNA community composition based on the Sørensen dissimilarity index. Points represent individual samples, with red indicating no‐rainfall samples and blue indicating rainfall samples. Environmental vectors fitted using “envfit” are shown as arrows; arrow direction indicates the gradient of the corresponding environmental factor, and arrow length reflects its correlation strength with community variation. Only significant factors (*p* < 0.05) are shown.

Figure 5 shows the result of PCoA. PCoA based on the Sørensen index showed that the first two axes explained 40.78% of the variation in community composition in the Mizunashi River. The two groups did not form distinct clusters in the principal coordinate analysis plot. Envfit analysis indicated that river discharge (*r* ^2^ = 0.60, *p* = 0.002), filtration volume (*r* ^2^ = 0.52, *p* = 0.019), and rainfall (*r* ^2^ = 0.60, *p* = 0.020) were significant drivers of community structure. In the Oka River, the first two PCoA axes explained 43.74% of the variation, and similarly, no distinct clustering was observed between the two groups. Envfit analysis also revealed that filtration volume (*r* ^2^ = 0.47, *p* = 0.029) and rainfall (*r* ^2^ = 0.81, *p* = 0.017) were significant drivers of community structure. The Tagami River exhibits a different pattern from the other two rivers. The first two PCoA axes explained 42.36% of the variation, and the two groups of samples formed a distinct separation in the PCoA plot. Envfit analysis identified discharge (*r* ^2^ = 0.56, *p* = 0.030), filtration volume (*r* ^2^ = 0.89, *p* = 0.030), and rainfall (*r* ^2^ = 0.52, *p* = 0.037) as significant drivers of community structure.

Table 3 presents PERMANOVA results, which supports the findings obtained from the PCoA. In the Mizunashi River, filtration volume had a significant effect on *β*_sor_ (*R* ^2^ = 0.14, *p* = 0.047). In the Oka River, filtration volume significantly affected *β*_sor_ (*R* ^2^ = 0.15, *p* = 0.038). In the Tagami River, filtration volume had a significant influence on both *β*_sor_ (*R* ^2^ = 0.23, *p* = 0.004) and *β*_sne_ (*R* ^2^ = 0.30, *p* = 0.001). In contrast, the effects of rainfall were relatively weak, with a significant impact observed only for *β*_sor_ in the Tagami River (*R* ^2^ = 0.15, *p* = 0.046). When the filtration volume was standardized, rainfall still had a significant effect (*R* ^2^ = 0.23, *p* = 0.041).

**TABLE 3:** Permutational Multivariate Analysis of Variance (PERMANOVA) results were based on rain (presence/absence of rainfall) as a categorical variable and volume (filtration volume) as a continuous variable.

	*β*_sor_	*R* ^2^	*p*	*β*_sim_	*R* ^2^	*p*	*β*_sne_	*R* ^2^	*p*
Mizunashi	Rainfall	0.06	0.774	Rainfall	0.08	0.444	Rainfall	0.08	0.713
	Volume	0.14	**0.047**	Volume	0.15	0.060	Volume	0.10	0.349
Oka	Rainfall	0.09	0.507						
	Volume	0.15	**0.038**						
Tagami	Rainfall	0.15	**0.046**	Rainfall	0.13	0.205	Rainfall	0.12	0.204
	Volume	0.23	**0.004**	Volume	0.09	0.751	Volume	0.30	**0.001**
Tagami (One liter)	Rainfall	0.23	**0.041**	Rainfall	0.24	0.081	Rainfall	0.30	0.116

*Note:* Bold values indicate *p* < 0.05. “Tagami (One liter)” refers to PERMANOVA results conducted only on samples with a filtered volume of 1000 mL.

## 4 Discussion

### 4.1 eDNA Metabarcoding

Using eDNA metabarcoding, we investigated the effects of rainfall on the detection of different vertebrate taxonomic groups by collecting river water samples with varying patterns following rainfall events. A total of 57 taxa, including fishes, mammals, birds, and amphibians, were detected in the 33 samples collected from the three rivers. For wild boars ( *S. scrofa* ), the 12S‐V primer used in this study could not distinguish between wild and domestic pigs (Harper et al. 2020). Although both types could exist in the study area, this study focused on the overall ecological patterns; therefore, the related sequences were conservatively assigned to the species level ( *S. scrofa* ). Future studies should consider using specific primers to differentiate between wild boar and domestic pigs. In the Tagami River, one ASV was identified as *Rana zhenhaiensis* . However, the likelihood of this species occurring locally is extremely low, as it is distributed only in China. Therefore, the sequence is more likely to be derived from *Rana japonica* .

### 4.2 RDI

The lowest total RDI value was observed during rainfall events, likely driven by a combination of factors, including reduced filtration volume caused by filter clogging, dilution of eDNA by rainfall, and PCR inhibition, which weakened the detectable eDNA signals. This effect was particularly evident for fish. Fish eDNA primarily originates from aquatic environments, and the dilution effect of rainfall is unlikely to be compensated by external eDNA inputs. Furthermore, filter clogging reduces the filtration volumes, further limiting the detection of fish eDNA. Accordingly, fish consistently exhibited the highest RDI values during periods without rainfall. However, the number of fish taxa detected in the Tagami River increased after the continuous rainfall. This may be due to sediment resuspension, which releases eDNA from benthic fishes, such as members of the family Gobiidae, into the water column, promoting detection. Previous studies have suggested that sediment is a more suitable substrate than water for fish eDNA detection (Sakata et al. 2021). However, because the 12S rRNA marker used in this study has limited taxonomic resolution, it remains unclear whether these newly detected taxa were benthic, midwater, or surface‐dwelling fish.

The highest RDI values for amphibians and mammals were observed during rainfall events. This may be because surface runoff transports terrestrial eDNA, replenishing the diluted portions (Condachou et al. 2025) and offsetting limitations imposed by smaller filtration volumes. Considering the behavioral characteristics of amphibians, the increase in RDI may not be entirely attributed to surface runoff. Amphibians tend to be more active after rainfall and are likely to release greater amounts of eDNA (Robinson et al. 2022), which explains why they were almost exclusively detected after rainfall events. However, continuous rainfall may cause differences between mammals. The increase in the number of detected mammalian taxa in the Mizunashi River after continuous rainfall may be explained by the predominance of livestock, whose eDNA sources were abundant near the sampling sites. Surface runoff likely transported this terrestrial eDNA into the river, thus increasing its detection rate during the second sampling event. In contrast, mammals detected in the Oka River were mainly wild animals with limited eDNA sources. As a result, the dilution effect of continuous rainfall may have exceeded the eDNA input from runoff, leading to fewer detections.

In the GLMM results, river discharge had a significant negative effect on the detection number. This was likely because the increased discharge during rainfall diluted the eDNA concentrations (Curtis et al. 2021), thereby reducing the detection number. In contrast, the filtration volume had a significant positive effect on the detection number because filtering larger volumes of water captured more eDNA (Ruan et al. 2022), leading to more detection counts. Rainfall itself did not significantly affect the number of detections. This may be because we could not determine when rainfall began to influence detection. Therefore, we used the total rainfall within 3 h prior to sampling as the environmental variable, although the actual time may differ.

### 4.3 Community Structure

Among the samples collected during the same period, the highest Sørensen dissimilarity indices were consistently observed in the Mizunashi River and Oka River in June 2023, and in the Tagami River in November 2022. These high dissimilarities were primarily driven by *β*_sim_. Rather than directly reflecting the effects of rainfall, these patterns were likely caused by filter clogging induced by rainfall, which resulted in the lowest filtration volumes for these sampling events. Compared to samples with larger filtration volumes, those with lower filtration volumes exhibited greater variability in terms of species composition (Wang et al. 2025), and the Sørensen dissimilarity index could be affected by low DNA concentrations or the non‐detection of certain taxa in some samples (Chase et al. 2011).

Under identical filtration volume conditions, *β*_sor_ in the Mizunashi River and the Oka River were nearly identical between rainfall and non‐rainfall periods. In contrast, in the Tagami River, the *β*_sor_ during rainfall events was lower than that under non‐rainfall conditions and was mainly driven by *β*_sim_. These results were further supported by PCoA and PERMANOVA analyses. In the Mizunashi River and the Oka River, rainfall did not significantly affect *β*_sor_, *β*_sim_, or *β*_sne_. Filtration volume significantly affected *β*_sor_ but had no significant effects on *β*_sim_ or *β*_sne_. Although filtration volume is an important factor influencing *β*_sor_ (Kawakami et al. 2023), the large variation in filtration volume among samples in the Mizunashi River and Oka River may obscure the effect of rainfall. This is supported by the results obtained from the Tagami River, where the filtration volume was relatively consistent, indicating that both filtration volume and rainfall significantly affected *β*_sor_, with filtration volume also significantly affecting *β*_sne_. Among replicate samples, *β*_sor_ was primarily driven by *β*_sne_ when differences in filtration volume were small, whereas *β*_sim_ became the dominant contributor to *β*_sor_ when differences in filtration volume were large. These findings suggest that within the same river system, the influence of filtration volume may outweigh that of rainfall, highlighting the importance of filtration volume in the sampling design for species detection. Furthermore, when the filtration volumes were equivalent, evaluating only the effect of rainfall revealed that the species composition tended to be more homogeneous during rainfall events (Condachou et al. 2025). This could be attributed to the lower water temperatures and reduced ultraviolet radiation following rainfall events, which favor the preservation of eDNA in the water columns (Strickler et al. 2015). Owing to the limited number of samples, further studies using larger sample sizes and standardized filtration volumes are required to verify the statistical significance and generalizability of this trend. However, rapid filtering of large volumes of turbid water remains a challenge (Baudry et al. 2024).

Comparisons across different watersheds further highlighted the importance of rainfall and filtration volume. During the first sampling event in May 2022, the *β*_sor_ values were similar between the Mizunashi River and the Oka River, despite considerable variation in the filtration volumes among the Oka River samples. In September 2022, when a complete 1‐L volume was filtered in both the Oka River and the Tagami River, the *β*_sor_ values were almost identical. In November 2022, the *β*_sor_ in the Tagami River was much higher than that in the Mizunashi River owing to insufficient filtration volume.

### 4.4 Other Effects on Detection Results

Because birds exhibit pronounced seasonal migration, there are large differences in community composition between seasons. Therefore, most bird taxa occur only in specific seasons and are difficult to detect continuously throughout the sampling period, resulting in generally low detection frequencies across all samples. For this reason, birds were not included in the statistical analyses presented above. Nevertheless, to provide additional ecological context, we explored the potential effects of rainfall on bird eDNA detection by comparing samples collected after the same time intervals (non‐rainfall: November 28, 2022; post‐rainfall: November 29, 2022).

Although the filtration volume of the post‐rainfall samples was lower (350–690 mL), the number of detected bird taxa was still higher than that during the non‐rainfall period (1000 mL). Under non‐rainfall conditions, only waterbirds were detected, whereas under rainfall conditions, both waterbirds (*Anatidae* sp. and *Gallinula chloropus* ) and non‐waterbirds (*Passeriformes* sp. and *Gallus gallus* ) were detected. One possible explanation is that airborne bird eDNA may enter rivers through rainfall (Lynggaard et al. 2024), whereas eDNA contained in bird droppings may also be transported into rivers via surface runoff after rainfall (Ragot and Villemur 2022), thereby increasing the opportunity for bird eDNA to enter rivers and improve its detection rate. Therefore, rainfall may promote the input of bird eDNA and increase its detection rate. However, seasonal community turnover remains the main factor affecting bird detection results.

Fish and amphibians exhibit changes in their spatial distribution and physiological status owing to seasonal variations, thereby affecting eDNA release. Many fish species (e.g., *Anguilla japonica* , *Plecoglossus altivelis* ) migrate between freshwater and marine environments during the breeding season, whereas amphibians typically enter aquatic habitats in large numbers during the breeding period for reproduction. Although such behaviors do not cause their DNA to disappear completely from aquatic environments, individuals at different life history stages show significant differences in DNA shedding rates. Studies have shown that juveniles generally release less DNA than adults (Maruyama et al. 2014; Ostberg and Chase 2022); therefore, when juvenile individuals dominate a population, eDNA concentrations in the environment may be relatively low. In addition, rainfall can create a dilution effect, further reducing eDNA detectability.

Owing to the discontinuation of the DNeasy PowerSoil Kit during the two non‐rainfall events, we used the DNeasy PowerSoil Pro Kit for the second non‐rainfall sampling. In the Mizunashi and Tagami Rivers, DNA extraction based on PowerSoil Pro resulted in greater detection, whereas the original PowerSoil Kit produced more detections in the Oka River. However, owing to the limited number of samples and other factors, we were unable to analyze the effects of differences in the extraction reagents.

One limitation of this study is that although all sampling, filtration, and DNA extraction equipment were sterilized to minimize the risk of contamination, field blanks, filtration blanks, and extraction blanks were not amplified or sequenced. The negative control included in the workflow was a PCR blank containing nuclease‐free water. Blanco et al. (2025) evaluated 87 eDNA studies and reported that nine did not include any negative controls and only one incorporated negative controls throughout all key stages, including field sampling, filtration, DNA extraction, PCR amplification, and sequencing. Among the different types of negative controls, PCR blanks were the most commonly used. Overall, negative controls are valuable not only for identifying potential sources of exogenous DNA contamination during sampling, filtration, extraction, amplification, and sequencing, but also for distinguishing true biological signals from background experimental noise, thereby reducing the risk of false‐positive detections. Therefore, we recommend that future studies include and analyze negative controls at critical stages of the workflow, including field sampling, filtration, DNA extraction, PCR amplification, and sequencing, to further improve the reliability of the eDNA data and the robustness of the results.

## 5 Conclusions

This study demonstrated that river water collected following rainfall events can be used to simultaneously detect aquatic and terrestrial species and evaluated how rainfall patterns and watershed characteristics influence eDNA detection. The results suggested that rainfall influenced the detectability of different taxonomic groups by altering multiple processes such as eDNA input, dilution, and resuspension. Although rainfall events could reduce the overall detection efficiency, particularly for fish, they could also enhance the detectability of terrestrial and semi‐aquatic species. The detection outcomes were further shaped by factors such as filtration volume and species‐specific ecological traits. Therefore, optimizing the sampling design based on the ecological characteristics of the target species is crucial for improving the detection efficiency in species‐specific eDNA studies. In addition, our findings highlight the importance of implementing multi‐timepoint sampling strategies across varying hydrological conditions and climatic events to achieve a more comprehensive assessment of watershed biodiversity.

## Author Contributions

**Chen Xu:** formal analysis (lead), investigation (equal), methodology (supporting), project administration (supporting), visualization (lead), writing – original draft (lead), writing – review and editing (equal). **Kei Nukazawa:** formal analysis (supporting), funding acquisition (lead), investigation (equal), methodology (lead), project administration (lead), visualization (supporting), writing – review and editing (equal).

## Funding

This work was partially supported by the Ministry of Education, Science, Sports and Culture through a Grant‐in‐Aid for Scientific Research (grant numbers 24H00329, 25K00041, and 25H01201).

## Conflicts of Interest

The authors declare no conflicts of interest.

## Supporting information


**Data S1:** Complete results of eDNA metabarcoding detection.

**Table S1:** PCR conditions.

## Data Availability

Raw reads generated during the current study are available in the DDBJ Sequence Read Archive (DRA) under the accession numbers DRA023808. Benefit Sharing: This study did not involve direct contact with animals in the survey area, particularly wild or endangered species. We are committed to investigating and detecting biodiversity without direct interaction with natural fauna.
